# Orthotopic Heart and Combined Heart Liver Transplantation: the Ultimate Treatment Option for Failing Fontan Physiology

**DOI:** 10.1007/s40472-021-00315-4

**Published:** 2021-02-04

**Authors:** Leigh C. Reardon, Jeannette P. Lin, Glen S. VanArsdell, Fady M. Kaldas, Gentian Lluri, Weiyi Tan, Katrina M. Whalen, Daniel Cruz, Ali Nsair, Mario C. Deng, Melissa A. Moore, Hillel Laks, Reshma M. Biniwale, Sammy Saab, Andrew Baird, James M. Wilson, Lorraine N. Lubin, Jure Marijic, Tiffany M. Williams, Christopher L. Wray, Joseph S. Meltzer, Vadim Gudzenko, Wolf B. Kratzert, Jacques Neelankavil, Robert S. Venick, Jamil A. Aboulhosn

**Affiliations:** 1 Ahmanson/UCLA Adult Congenital Heart Disease Center, Division of Adult Cardiology, 100 UCLA Medical Plaza Suite 630E, Los Angeles, CA 90095 USA; 2 UCLA Children’s Heart Center, Division of Pediatric Cardiology, 200 UCLA Medical Plaza Suite 330, Los Angeles, CA 90095 USA; 3 UCLA Department of Surgery, Cardiothoracic Surgery, 100 UCLA Medical Plaza Suite 630E, Los Angeles, CA 90095 USA; 4 UCLA Department of Surgery, Pfleger Liver Institute, 200 UCLA Medical Plaza Suite 214, Los Angeles, CA 90095 USA; 5 Ahmanson/UCLA Cardiomyopathy Center, 100 UCLA Medical Plaza Suite 630E, Los Angeles, CA 90024 USA; 6 UCLA Department of Medicine, Pfleger Liver Institute, 200 UCLA Medical Plaza Suite 214, Los Angeles, CA 90095 USA; 7 UCLA Department of Nephrology 100 UCLA Medical Plaza Suite 690,, Los Angeles, CA 90095 USA; 8 UCLA Department of Anesthesiology and Perioperative Medicine, 757 Westwood Plaza, Suite 3325-7430UCLA, Los Angeles, CA 90095 USA; 9 Department of Pediatric, Gastroenterology, 200 UCLA Medical Plaza Suite 265, Los Angeles, CA 90024 USA

**Keywords:** Fontan, Single ventricle, Liver transplant, Heart transplant, Enteropathy, Embolization

## Abstract

**Purpose of the Review:**

This is a comprehensive update on failing Fontan physiology and the role of heart and combined heart and liver transplantation in the current era.

**Recent Findings:**

Single ventricle physiology encompasses a series of rare congenital cardiac abnormalities that are characterized by absence of or hypoplasia of one ventricle. This effectively results in a single ventricular pumping chamber. These abnormalities are rarely compatible with long-term survival if left without surgical palliation in the first few years of life. Surgical treatment of single ventricle physiology has evolved over the past 60 years and is characterized by numerous creative innovations. These include the development of arteriopulmonary shunts, the evolution of partial cavopulmonary connections, and the eventual development of the “Fontan” operation. Regardless of the type of Fontan modification, the long-term consequences of the Fontan operation are predominantly related to chronic central venous hypertension and the multi-organ consequences thereof. Atrial arrhythmias can further compromise this circulation.Patients with single ventricle physiology represent a special sub-segment of congenital cardiac transplants and are arguably the most challenging patients considered for transplantation.

**Summary:**

This review describes in detail the challenges and opportunities of heart and liver transplantation in Fontan patients, as viewed and managed by the experienced team at the Ahmanson/UCLA Adult Congenital Heart Center.

## Background

**Fig. 1-:**
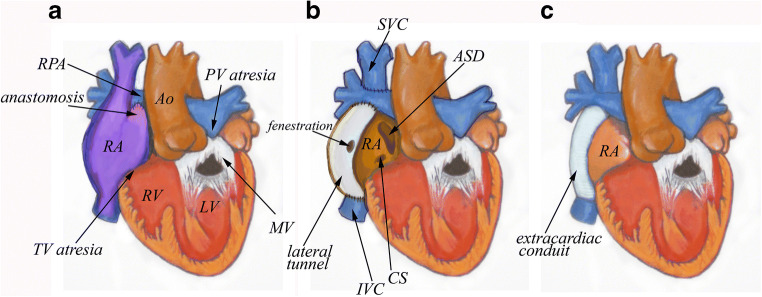
Major Fontan subtypes- A) The early era of the Fontan operation included the right atrium (RA) within the Fontan circuit, usually with the right atrial appendage used as an anastomosis between the RA and right pulmonary artery (RPA). These types of RA-PA Fontan operations were performed in the 1970’s and 1980’s and were supplanted in the 1990’s by the lateral tunnel Fontan (B) which often included a small fenestration. It consists of an intra-atrial baffle from the inferior vena cava (IVC) to the RPA. The superior vena cava (SVC) is connected directly to the RPA (Glenn shunt), hence this is a total cavopulmonary connection that still includes the posterior wall of the right atrium within the Fontan circuit. C) The extra-cardiac Fontan is the most widely performed Fontan sub-type since the late 1990’s and consists of a Gore-tex conduit from the inferior vena cava to the RPA, the right atrium is not included in the Fontan circuit

Single ventricle physiology encompasses a series of rare congenital cardiac abnormalities that are characterized by absence of or hypoplasia of one ventricle. This effectively results in a single ventricular pumping chamber. These abnormalities are rarely compatible with long-term survival if left without surgical palliation in the first few years of life. Surgical treatment of single ventricle physiology has evolved over the past 60 years and is characterized by numerous creative innovations. These include the development of arteriopulmonary shunts, the evolution of partial cavopulmonary connections, and the eventual development of the “Fontan” operation. The early forms of the Fontan procedure consisted of a connection between the right atrium and the pulmonary artery (Fig. 1A). These early Fontan subtypes included all, or nearly all of the right atrial body. These were abandoned by the early 1990’s due to high rates of atrial arrhythmias and inefficient hemodynamics in favor of the more streamlined lateral or intra-atrial tunnel Fontan types. These subtypes include a modification that allows for Fontan pressure modulation via a small fenestration (Fig. 1B). The Fontan tunnel excludes much of the right atrium from the increased Fontan pressure and is, therefore, less pro-arrhythmic. This modification also decreases blood flow turbulence due to the cylindrical shape of the tunnel. The extra-cardiac Fontan conduit is currently the most widely utilized Fontan modification (Fig. 1C) due to the ease of the operation compared to the lateral tunnel modification, the need for fewer atrial suture lines, and the complete exclusion of atrial tissue from the Fontan conduit.

Regardless of the type of Fontan modification, the long-term consequences of the Fontan operation are predominantly related to chronic central venous hypertension and the multi-organ consequences thereof. A ‘typical’ Fontan pressure in an adult is 10–14 mmHg,^1^ which is more than double the normal central venous pressure of an individual with a biventricular circulation. There are inevitable further venous pressure elevations with exercise, or with any conditions that could increase pulmonary arterial resistance. The essential problem is that there is only one functional ventricle and, therefore, flow to the pulmonary arterial bed is via passive cavopulmonary connections that are highly dependent on respiratory mechanics and peripheral muscular assistance to maintain adequate cardiac output.

Atrial arrhythmias can further compromise this circulation, especially in those that have older RA-PA Fontan subtypes, the majority of these patients eventually suffer from atrial arrhythmias; and although patients with more modern types of Fontans are less likely to have sustained atrial arrhythmias, the arrhythmia risk increases appreciably as these patients age.^2,3^ There are other consequences to this passive circulation including hepatic venous hypertension resulting in liver congestion, progressive fibrosis, cirrhosis and the increased likelihood of hepatocellular carcinoma.^4^ Chronic mesenteric edema can result in protein loss and malabsorption. Peripheral venous hypertension results in venous stasis and varicosities. Fontan physiology is characterized by a chronic low cardiac output state with limited reserve to increase the cardiac output during exercise. The hemodynamic characteristics of various types of Fontan failure are demonstrated in Table 1 and can be broadly divided into those with reduced vs normal single ventricle systolic function.^5^ One additional subtype that is emerging among adult patients with advanced Fontan-associated liver disease (FALD), is the Fontan patient in whom the cardiac output can be elevated due to a high output and low systemic arterial resistance state encountered with liver failure and decompensated cirrhosis.^6^

**Table 1:** Hemodynamic characteristics of the various types of commonly encountered Fontan failure (FF)

	Systolic function	Ventricular diastolic pressure	Cardiac output	SVR	PVR
FF-reduced EF	reduced	elevated	low	high	normal
FF-normal EF	normal	normal/low	low	normal/high	normal/high
FF- liver failure	normal or reduced	elevated	normal/high	normal/low	normal/high

Traditional heart failure medical therapies and rhythm control measures have demonstrated limited success in patients with the RA-PA Fontan. These patients are often considered for surgical Fontan conversion to an extracardiac conduit with concomitant arrhythmia surgery and epicardial pacemaker implantation.^3,7,8^ While many patients undergoing Fontan revisions have favorable arrhythmia control and improved hemodynamics, the morbidity and mortality of this intervention in some series approaches 20%.^3,9^ With improved orthotopic heart transplant (OHT) survival, Fontan conversions have largely fallen out of favor at our institution. Congenital heart disease specialists should also address issues that may be contributing to heart failure in the Fontan patient such as addressing areas of stenosis in the Fontan circuit, reducing the number of arterial collaterals that might contribute to volume overload on the single ventricle, and intervening on significant atrioventricular valve regurgitation when feasible.

Patients with single ventricle physiology represent a special sub-segment of congenital cardiac transplants and are arguably the most challenging patients considered for transplantation. Transplantation in patients with Fontan physiology is often viewed as carrying high or prohibitively high risk given reports of poor survival. As recently as 2009, Lamor et al. reported a 1-year post-transplant survival of 71% in Fontan patients and Davies et al. (2012) examined 43 patients with failing Fontan physiology who had a 90-day post-transplant survival of only 65%.^10,11^ Similarly, Tabarsia et al. performed a meta-analysis of 12 retrospective studies from 1995 to 2015 that showed a one-year post-transplant survival of Fontan patients of 80.3%, and this in a young cohort of in patients aged 7 to 24 years.^12^

While the complexity and risk of OHT and combined heart and liver transplantation (CHLT) should not be downplayed, more favorable outcomes in the last 5 years have shifted our understanding of OHT and CHLT in the Fontan patient and is likely due to the following factors: Improved multi-disciplinary patient selection, improved surgical planning and management, pre-transplant optimization, intraoperative congenital cardiac anesthesia care, cardiac intensive care management and post-transplant care with coordination of care teams. Shi et al. reported the Australia and New Zealand experience of 34 patients with a median age of 17 (11–31) years having a one-year survival of 91%.^13^ Additionally, a multi-institutional study published in April 2017 of 38 Pediatric Heart Transplant Study (PHTS) centers from 1993 to 2014 showed better outcomes in a contemporary era analysis. When looking at 402 Fontan patients (<18 years of age) who underwent heart transplant—patients in the early era of transplant (1993–2006) had a 1-year post-transplant survival of 77% and a more contemporary era (2007–2014) had a 1-year post-transplant survival of 89%.^14^

Recently, attention has focused on adults (>18 years of age) as a distinct group of Fontan patients that have faced the adverse consequences of the Fontan circulation for a longer time than their pediatric counterparts. Interestingly, there may be a survival benefit or resiliency to surviving to adulthood with single ventricular physiology and then undergoing OHT or CHLT. Menachem et al. reported a series between 2010 and 2016 at the University of Pennsylvania that included 20 adult congenital heart disease patients of which 8 patients had failing Fontan physiology. The only mortality in their cohort was of a non-Fontan patient.^15^ Our center, the Ahmanson/UCLA Adult Congenital Heart Disease Center, reported in 2018 a series of 20 adult patients with Fontan palliation −15 of whom underwent OHT and 5 of whom underwent CHLT – with a 100% one-year survival and single mortality at 3 years post-transplant secondary to severe coronary artery vasculopathy.^16^ Since the publication, two patients in this cohort have died – one patient died due to a rejection episode at 2.1 years post-transplant and another patient died of restrictive cardiac physiology at 2.9 years post-transplant. Subsequent to the 2018 publication, our center has completed an additional 5 OHT and 8 CHLT with a single peri-operative mortality due to uncontrolled bleeding. Vaikunth et al. from Stanford also published their experience with en-bloc CHLT in 9 patients with a median age of 20.7 years (range of 14.2–41.3) with a 100% 1-year post-transplant survival.^17^

This review will explore some of the strategies that our institution has employed in the selection and management of the Fontan patient undergoing heart (OHT) or combined heart liver transplant (CHLT).

### Timing and Type of Transplantation

Timing for OHT or CHLT in patients with single ventricle physiology is problematic given the variability of presentation. Despite this, early referral to a center with multidisciplinary experience in the management of complex congenital heart disease provides the patient with the best opportunity for a successful outcome rather than being deemed ineligible for transplant secondary to advanced disease. Many patients are referred from out-of-state and may present with extensive insurance, financial and social support challenges which take time to address by the multidisciplinary team. Well-developed programs that perform a relatively large volume of CHD transplants may come to very different conclusions about the timing and candidacy for transplantation, further emphasizing the need for early referral. In addition, there is improved wait list outcomes for ACHD patients that are listed at programs that have comprehensive care center accreditation through the Adult Congenital Heart Association (ACHA).^18^

Applying the typical OHT listing guidelines to Fontan patients may be problematic, but they can serve as a useful reference point – for example a VO2 max of <14 ml/kg/min is a listing guideline for OHT in those with a biventricular circulation. Indices for exercise tolerance are reduced in most patients with a Fontan with the average well-compensated patient having a VO2 max of around 24 ml/kg/min. In a Fontan patient, one must not only consider the functional limitations of the individual patient but also the multi-organ consequences of failing Fontan physiology. Close follow up and our accumulating experience in trending changes in functional capacity and end organ function will, hopefully, allow us to time OHT before irreversible changes in the liver may necessitate CHLT.

There is no clear consensus on how to define the failed Fontan. At the Ahmanson/UCLA Adult Congenital Heart Disease Center, we define failing Fontan physiology in the following way: dysfunction of the Fontan circulation resulting in reduced functional capacity, volume overload, recurrent/persistent arrhythmias, and/or multi-organ dysfunction. Etiologies may vary and multiple concomitant processes may manifest including ventricular dysfunction, Fontan pathway obstruction, valvular failure, elevated pulmonary vascular resistance, lymphatic insufficiency, advanced liver disease and persistent arrhythmias.

There is no clear consensus for when transplant evaluation should be initiated in the Fontan patient and there are no published guidelines in this patient population. Delaying OHT until the Fontan patient has developed hepato-renal failure clearly leads to worse outcomes with or without transplantation. Delayed transplantation may have contributed to some of the poor outcomes reported in the earlier OHT studies referenced in the background section. Therefore, it stands to reason that Fontan patients that continue to manifest severely reduced functional capacity, volume overload, persistent arrhythmias, protein losing enteropathy, and/or multi-organ dysfunction despite optimal medical/interventional/electrophysiologic management should be referred for heart or multi-organ transplantation evaluation. Increased symptoms and continued decline in functional capacity despite optimal medical management is another sign that consideration should be given to transplant evaluation.

The decision as to whether a patient may benefit from single or multi-organ transplantation is similarly challenging and fraught with little data to support or refute any given approach. The most commonly encountered scenario in Fontan patients being considered for transplant is the inevitable presence of some degree of liver fibrosis with most patients demonstrating ‘cirrhotic’ changes on imaging, although it has been demonstrated that cirrhosis on biopsy is less common and often does not correlate well with imaging modalities such as CT and ultrasound.^19^ Patients with cirrhosis, regardless of the etiology may have complicated cardiopulmonary bypass courses and poor outcomes when undergoing cardiac surgical procedures.^20^ The finding of cirrhotic changes on liver biopsy is a strong indication that a combined heart-liver transplantation should be considered. Other clinical/imaging manifestations of cirrhosis and stigmata of liver failure, such as lower esophageal varices, ascites, splenomegaly, thrombocytopenia are also taken into consideration.^21–23^ MELD-XI >17 has been demonstrated to be predictive of worse survival post-transplant in congenital heart disease patients but in reality is not a pivotal determinant in our selection process.^23^

Multiple prior operations in Fontan patients can result in restrictive lung disease due to impaired diaphragmatic function and fibrosis. Diaphragmatic function may range from mild decrease in diaphragmatic excursion to complete hemidiaphragm paralysis due to phrenic nerve injury. When undertaken soon after phrenic nerve injury, diaphragmatic plication has been demonstrated to improve respiratory function, though diaphragmatic plication in the patient with remote injury and a chronically elevated hemidiaphragm is of less clear benefit. Nonetheless, plication at the time of transplant for patients with borderline restrictive lung disease may be considered. Diaphragmatic atrophy, as a part of skeletal muscle mass loss, is associated with chronic liver disease and may contribute to failing Fontan patient symptoms and contribute to difficulties in weaning mechanical ventilator support in the post-operative stetting. Rarely, patients with protein losing enteropathy may also have a plastic bronchitis which carries significant peri-operative implications for oxygenation and separation from cardiopulmonary bypass after OHT or CHLT; therefore plastic bronchitis should be ruled out with chest imaging prior to listing. The use of perioperative veno-venous ECMO may be used to bridge these patients perioperatively as needed.

Nephrology evaluation is indicated in this population due to chronic low cardiac output and renal venous congestion. Baseline CKD is generally present in this patient population due to chronic heart failure, passive congestion, renal toxic medications and contrast exposure. Post-transplant, renal function often improves in patients with stage I-II CKD due to improvement in cardiac output; however, continuous renal replacement therapy (CRRT) is often needed in the immediate post-transplant period for optimization of fluid status.

Patients with Fontan palliation often have mild thrombocytopenia due to splenic sequestration, and mild leukopenia. Hematology assessment may be helpful to ensure that the patient does not have a primary bone marrow disorder. The baseline coagulation status of these patients should be delineated preoperatively and the post-surgical coagulopathy which is multifactorial and often profound.

A multi-disciplinary, closely integrated, and frequently communicative team is essential to any program that seeks to perform heart or multi-organ transplantation on failing Fontan patients (Table 2). We have found it extremely helpful to have in person or virtual meetings (a necessity during the COVID-19 pandemic) on a regular basis (typically ever 2–3 weeks) with the cardiac congenital/transplant surgical/medical teams, hepatology and liver transplant surgical teams and other medical consultants to discuss these patients in depth and review their imaging studies together. Reviewing a patient for OHT and CHLT in an initial core stakeholders meeting can take upwards of an hour or more per patient. We have found it vital to have a small group of dedicated team members familiar with the intricacies of a Fontan transplant spend time reviewing each patient and using each patient as a programmatic continual learning exercise. A separate focused, surgical meeting after acceptance in the full heart and/or liver committees to discuss the surgical/operative and perioperative plan is necessary for those with complex anatomy and those requiring multi-organ transplantation. At our institution, this meeting includes the cardiac and liver transplant surgical teams, cardiac and liver anesthesia teams, as well as the perfusionists and specifically addresses the technical steps involved in coordinating and orchestrating such a complex surgical undertaking.

**Table 2:** Required services/specialties for Fontan heart and liver transplantation

**Team/Specialty**	**Considerations/Perspectives**
Adult congenital heart disease cardiology	Congenital diagnosis, prior surgeries, anatomy and physiology
Congenital interventional cardiology	Invasive hemodynamic assessment, intervention to address residual venous or arterial stenosis, coil embolization collateral vessels to decrease bleeding during transplant
Congenital electrophysiology	Ablations, arrhythmia and device management
Congenital Radiology/Interventional Radiology (IR)	Provide detailed anatomic assessments and IR to obtain liver biopsies
Congenital/transplant cardiac surgery	Assess surgical risk and technical feasibility of heart or combined organ transplant
Cardiomyopathy/transplant cardiology	Heart failure optimization, MCS options, management of post-operative immunosuppression and surveillance for rejection
Transplant psychiatry	Screen/treat depression and anxiety
Transplant social worker	Assess social support and compliance
Transplant Financial	Assess insurance compatibility and recommend and coordinate necessary changes.
Transplant infectious disease	Management and prevention of opportunistic infections
Heart Transplant Nursing Coordination	Primary coordinator of complex evaluation with all team members from point of referral to transplant, patient education.
Immunogenetics	Evaluate patient antibody profiles
Pulmonology	Evaluate pulmonary function pre-transplant
Nephrology	Evaluate need for concurrent renal transplant; management of CKD, and perioperative dialysis for volume management
Hematology/Oncology	Evaluate chronic leukopenia and thrombocytopenia, rule out occult malignancy.
GI/Hepatology	Evaluate whether patient requires concurrent liver transplant, assessment of varcies
Liver transplant surgery	Evaluate suitability for concurrent liver transplant
Liver Transplant Nursing Coordination	Coordinate liver evaluation, patient education.
Cardiac and Liver Transplant Anesthesiology	Preoperative anesthesia evaluation, identifying modifiable risks an make detailed plan for perioperative anesthesia plan
Blood Bank	Prepare for massive transfusions

### Medical and Surgical Considerations

**Fig. 2:**
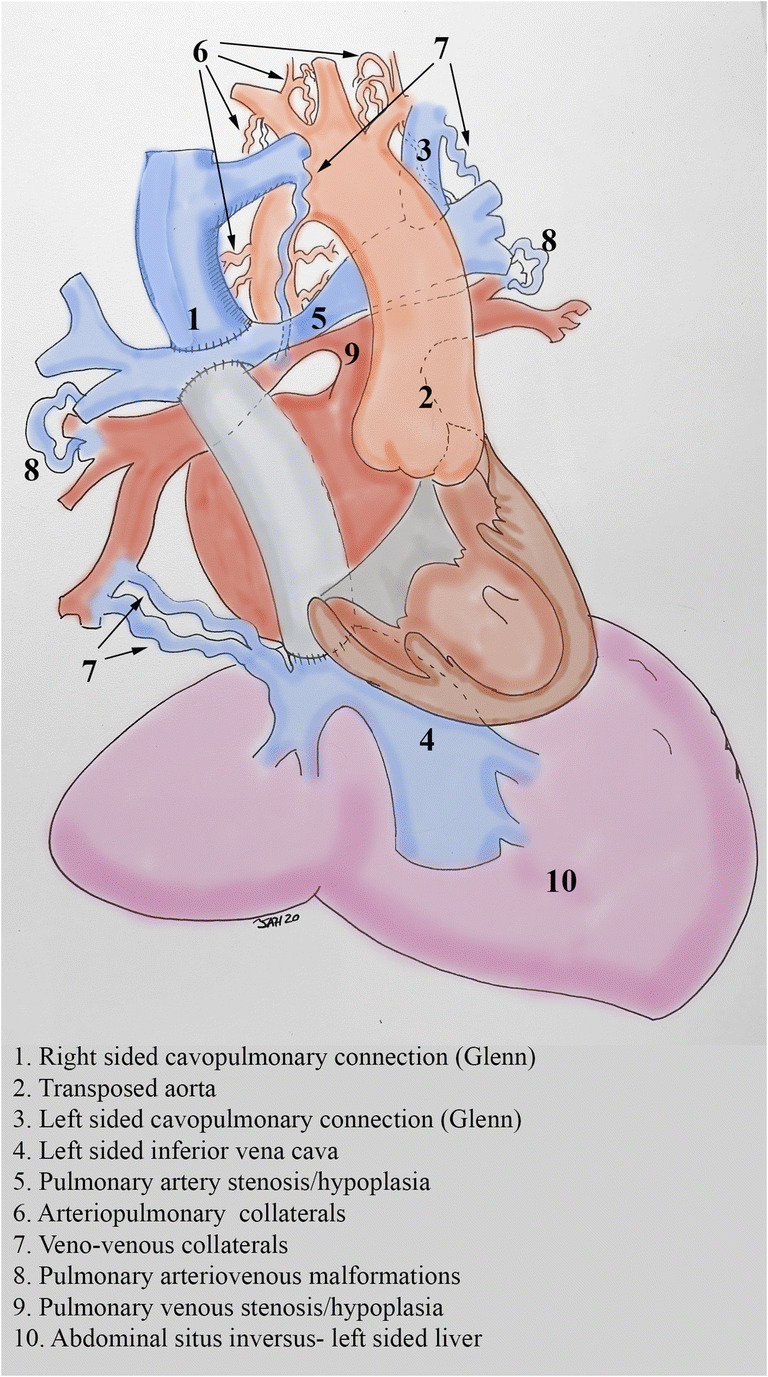
Anatomic and surgical considerations when considering heart or heart and liver transplantation in a patient with heterotaxy, transposition, total anomalous pulmonary venous connection (previously repaired), intra-atrial tunnel Fontan, left sided superior vena cava, numerous arterial and venous collaterals, bilateral Glenn shunts and pulmonary artery stenosis/hypoplasia. Every one of these factors requires special consideration and appropriate planning to ensure success. In selected complex cases using 3D printed models allows for advanced surgical planning and optimal repair of vascular anomalies

The Fontan operation is utilized to treat a variety of conditions that are characterized by a functional “single ventricle” and therefore is an operation that is superimposed on heterogeneous underlying anatomic substrates, from those with isolated tricuspid atresia with normal cardiac situs and looping (Fig. 1) to those with complex transposition, heterotaxy, and anomalous venous connections (Fig. 2). The manifestations of Fontan circulation failure can also be varied (Table 1). Therefore, it is clear that each failing Fontan patient is a unique entity and deserves consideration and treatment as such when evaluating the candidacy of a patient for OHT or CHLT, when planning surgical intricacies of OHT or CHLT, and when optimizing the patient once accepted for transplant.

**Fig. 3:**
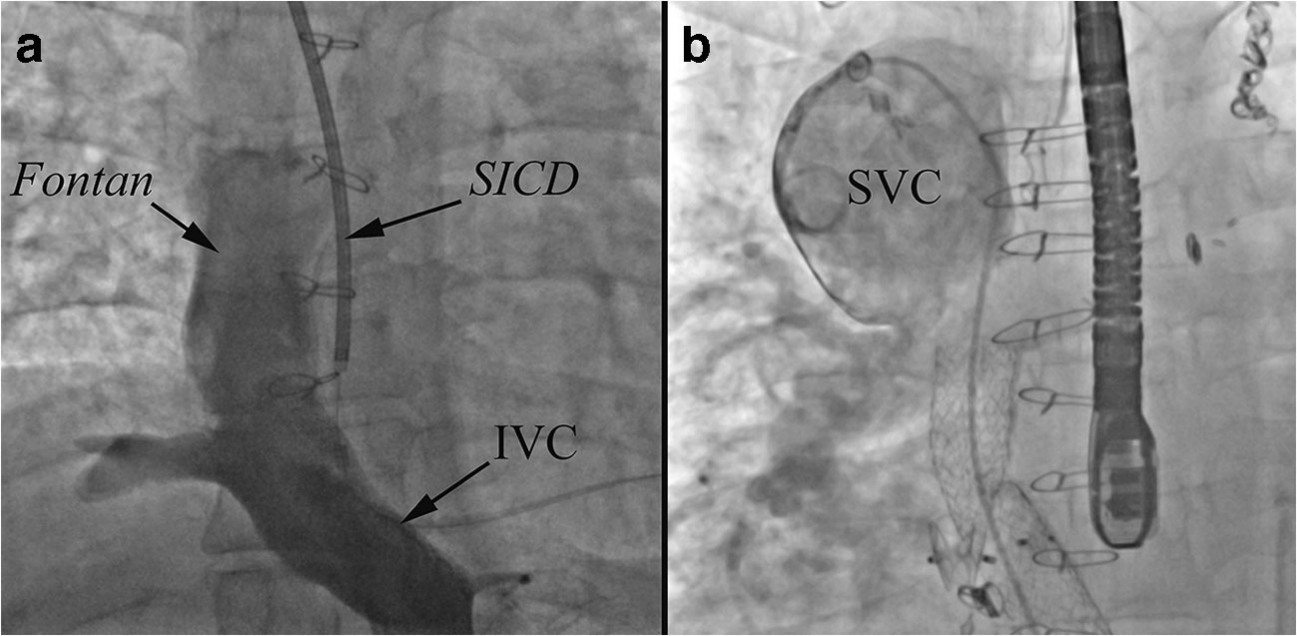
A. Inferior vena cava (IVC) angiography of a 39 year old male with heterotaxy and a lateral tunnel Fontan (same patient as in Fig. 2), left sided inferior vena cava (IVC) and a subcutaneous ICD (SICD). B. Superior vena cava (SVC) angiography in a 42-year-old male with an extracardiac Fontan and Glenn shunt, note the severe dilation of the SVC, this requires SVC reconstruction at the time of heart transplantation

The anatomic and surgical challenges are highlighted in Figs. 2 and 3. The surgical plan should consider and address each anatomic issue to ensure the appropriate organ size is selected and sufficient caval and arterial tissue is procured to allow for unobstructed connections. Typically, the surgical planning notes will make sure that the procurement surgeon knows to take ample/extended lengths of the IVC/SVC, aorta and pulmonary arteries from the donor when necessary. It can occasionally be difficult to procure extended lengths of the pulmonary arteries when the lungs are being procured for another recipient, but most congenital surgeons are experienced with pulmonary artery reconstructions. In fact, it is exactly these types of unique considerations that have prompted us to develop a program where the congenital cardiac surgeons are the transplant surgeons for Fontan patients.

When considering liver transplantation in a sequential or en-bloc fashion, abdominal visceral situs and inferior vena caval location are important factors. Each center considers the most effective approach when CHLT is needed – while there may be center specific considerations for en bloc versus sequential implantation the patient’s anatomy may ultimately play the determinant role. Our team has found that sequential implantation works best for our surgical teams and tends to afford less crowding on the operating table. Procurement of an en bloc heart liver dual organ block also requires experienced procurement surgeons as the diaphragm has to harvested along with the IVC to prevent injuries to phrenic veins that can cause significant bleeding post-implantation. Warm ischemia to the heart has to be avoided during the back-table liver dissection, especially if vascular conduits need to be constructed for an anomalous hepatic artery. This can be achieved by packaging the heart separately within the enbloc specimen to avoid exposure to room temperature.

### Preparation for Fontan Transplantation

All patients undergoing the transplant evaluation process will have a routine cardiac catheterization, upper endoscopy and liver biopsy. It is vital to determine hemodynamics, collateral burden, and addressable conditions such as a Fontan conduit stenosis, assess for varices and evaluate the degree of liver fibrosis. The endoscopy and liver biopsy are used to better evaluate patients and determine the level of liver dysfunction beyond simple laboratory evaluation since many patients will have preserved synthetic function while still having significant fibrosis or cirrhosis. If a patient has biopsy proven cirrhosis (as evidenced by extensive bridging fibrosis) we uniformly recommend a CHLT. Furthermore, our current practice is that patients with significant bridging fibrosis and stigmata of liver disease with varices and splenomegaly are referred for CHLT. Each patient is assigned a VAST score, which is calculated by the sum of clinical findings (Varices, Ascites, Splenomegaly, and Thrombocytopenia <150 K) and if >2 is a predictor of major adverse events in Fontan patients. The VAST score is factored in to the potential need for liver transplantation in those with evidence of portal hypertension and is presented during our multi-disciplinary meetings.^22^ All patients must have an up-to-date liver biopsy if cirrhosis has not already been demonstrated and CHLT decided upon, preferably within 6 months of listing. We routinely request that our liver pathologists re-review slides from outside referring institutions.

**Fig. 4:**
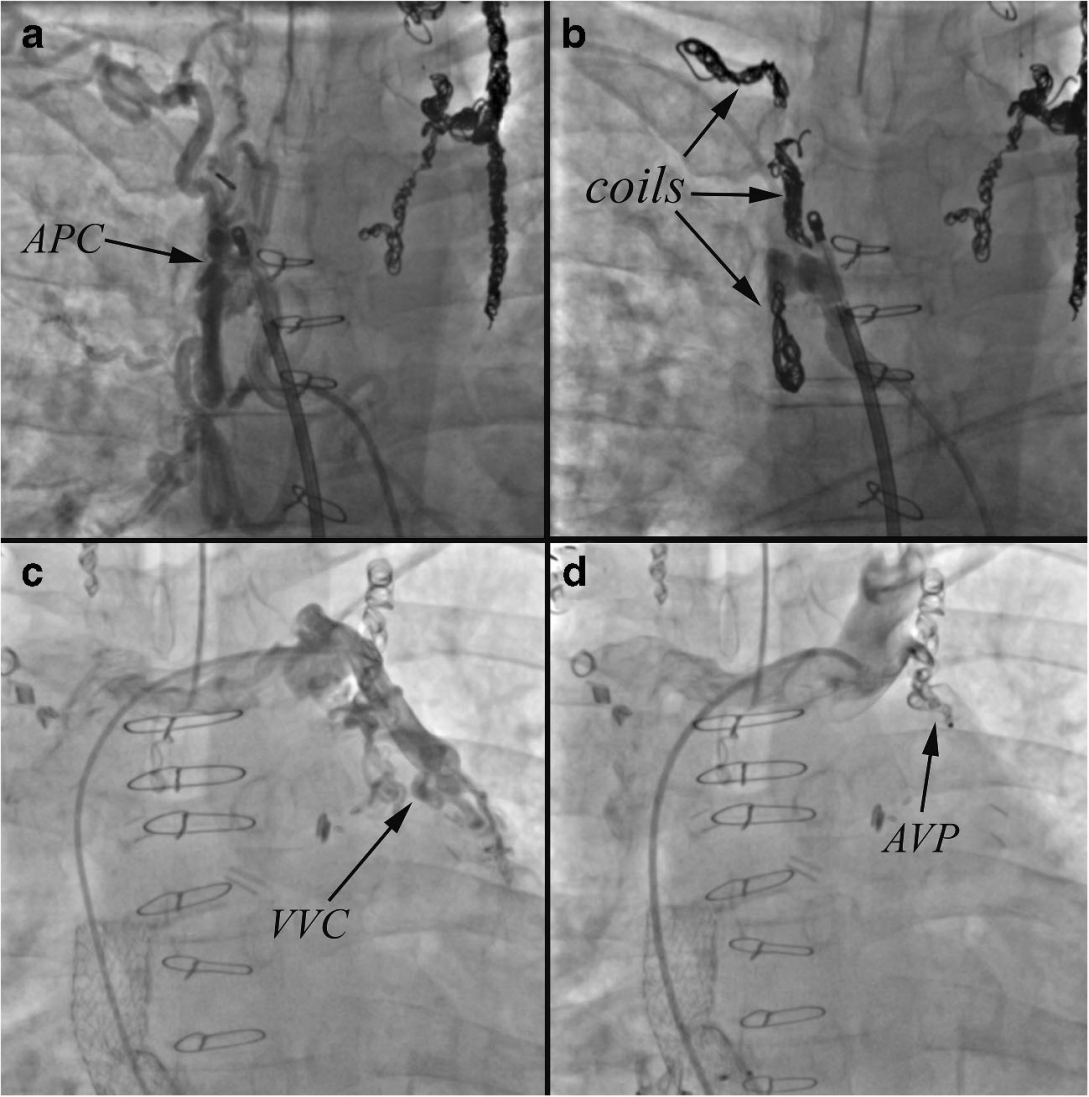
A. Selective angiography in the descending thoracic aorta of the 39 year old patient described in Figs. 2 and 3A demonstrates a large aortopulmonary collateral (APC) that required coil embolization (B) to decrease bleeding risk during transplantation. C. Veno-venous collateral from the innominate vein to the left sided pulmonary veins in the patient described in Fig. 3B. D. Amplatzer vascular plug implantation results in complete occlusion of flow. The venous collaterals could contribute to the heavy bleeding seen during Fontan transplantation and therefore the protocol at the Ahmanson/UCLA ACHD program is to occlude both arteriopulmonary and veno-venous collaterals prior to listing for transplantation

One of the unifying issues for post-transplant complications has been the degree of intra-operative and post-operative bleeding which is the single most important cause of intra- and peri-operative mortality. The difficult to control operative bleeding is commonly due to the presence of aortopulmonary (AP collaterals) and to a lesser extent venovenous (VV) collaterals. A strategy for targeted transcatheter coil embolization or device occlusion of VV and AP collaterals prior to transplantation should be considered to mitigate surgical risk (Fig. 4). Notably, occlusion of decompressing VV collaterals may increase Fontan pressure, and should be undertaken with caution, particularly if there is a chance that the patient may not be accepted for transplantation.^24^ We generally will perform embolization of AP collaterals first and only undertake VV collateral embolization if the patient is accepted for transplantation. Bleeding particularly on chest entry increases the complexity of the procedure, potentially increases donor organ ischemic times, and may necessitate significantly larger blood product transfusions during the transplant – thereby increasing the inflammatory response, further burdening the renal system, and potentially sensitizing the patient to alloantibodies. It is our practice, to schedule coil embolization catheterizations after the patient has been accepted for transplant and not to list until the majority of the AP and VV collaterals have been occluded. Most of our patients require more than one visit to the catheterization laboratory given the high burden of collaterals encountered and the importance of avoiding large iodinated contrast loads and excessive procedural radiation. Patients who are listed but have not received an organ will have repeat surveillance catheterization to assess for recurrent collateral burden every 3 months or as deemed necessary by clinical evaluation.

Some Fontan patients are highly sensitized to foreign antigens due to prior blood transfusions or use of cadaveric non-decellularized material during prior cardiac surgeries. Highly allosensitized patients face longer wait times as their antibodies preclude them from receiving organs from a substantial proportion of the donor pool. In addition, they are at increased risk for cardiac rejection, and may require more aggressive immunosuppression post-transplant. For such patients, the heart transplant cardiologist and immunogenetics team often consider a trial of desensitization therapy; if the antibody profiles improve with desensitization therapy, they may become acceptable candidates for transplant listing. Desensitization at our institution usually starts with intravenous immunoglobulin (IVIG), rituximab and plasmapheresis with progression to bortezomib as tolerated. For patients who are accepted for transplant listing in spite of high allosensitization, desensitization therapy can decrease their wait times by expanding their ability to accept organs from a larger proportion of the donor pool, and decrease risk of rejection post-transplant. Patients with PLE can have suspiciously low levels of antibodies, due to GI loss of protein including antibodies via their gastrointestinal tract. Post-transplant, a heightened level of surveillance for antibodies should be undertaken in all patients with a history of PLE.

### Post-Listing Optimization

At our center, once the patient has been accepted for OHT or CHLT, a surgical planning meeting as referenced above helps coordinate and prepare the team for the ensuing orchestration of the transplant. Our detailed written plans are disseminated to all team members help ensure maximal preparedness for the complex procedure, adequate time for induction of anesthesia, obtaining adequate vascular access to be able to support patient in cases of massive blood loss, safe chest entry, control of bleeding and explant of the recipient heart. Furthermore, the patient’s antibody profile and renal function are reviewed to determine the need for post-transplant induction therapy and the timing for oral immunosuppression. As a team we also use patient complexity to select the geographic range of acceptable donors in order to optimize the donor pool while also minimizing donor ischemic time. Typically we aim to keep donor travel time to the hospital to less than three hours. Donor height/weight parameters are adjusted to ensure optimal patient size matching, particularly for patients with heterotaxy, while not limiting available donors. We calculate donor predicted heart mass (PHM) at the time of an offer to ensure appropriate donor size matching and to avoid accepting undersized donor heart.^25^ While many centers empirically oversize donor hearts in attempt to compensate for underestimated pulmonary pressures in the Fontan circuit our practice has not followed this pattern and appears to be supported by a recent analysis by Clark et al.^26^ Balancing the benefits of a smaller donor with less vertical span in a restricted mediastinum with a larger donor with a more robust right ventricle that can cope with the aortopulmonary collateral flow and multi-unit transfusion is a difficult task. As a general rule the height of the donor determines the vertical dimensions of the donor heart, which will determine fit. The donor weight is a secondary consideration except in CHLT where the liver will be considerably oversized and will not be accommodated in an abdomen with a narrow subcostal arch (males) in the absence of ascites. It is important to assess each donor individually and use the recipient CT scan/echo dimensions to guage the fit of the new organ.

While awaiting transplant, ACHD patients with tenuous hemodynamic status or evidence of progressive end-organ damage may benefit from inpatient admission and listing, as this provides an opportunity for ongoing optimization prior to transplant. Continuous infusion of inotropes can improve organ perfusion, careful titration of diuretics can maintain the patient in a euvolemic state, thus mitigating risk at the time of transplant and possibly even reverse some of the end organ damage caused by venous congestion and low cardiac output. We closely monitor daily weights and Fontan pressure via a PICC line in order to closely titrate medical therapies. Effective preoperative optimization improves hemodynamic stability during induction of general anesthesia and in pre CPB period. This shortens CPB duration and reduces blood loss by allowing surgical teams to carefully dissect heart out without injuring diaphragm, mediastinal structures and obtain hemostasis before heparinization for CPB.

Under the UNOS adult listing criteria that were adopted in October of 2018, patients with a Fontan who are waiting at home are listed status 4 (Table 3). Patients who are listed and hospitalized are usually listed as status 3E or status 2E based on the criteria described in the UNOS Review Board (RB) Guidance for Adult Congenital Heart Disease (CHD) Exception Requests document.^27^ The review board guidance for exceptions are summarized in the Table 3 and take into account the anatomic inability to have continuous invasive hemodynamic monitoring in these patients. Most of our patients who are admitted for hemodynamic instability or end-organ damage are felt to be at high risk of decompensation, therefore, exception requests are initiated for these patients.

**Table 3:** Unified Network of Organ Sharing (UNOS) Review Board (RB) Guidance for Adult Congenital Heart Disease (CHD) Exception Requests^27^

Recommended criteria for ACHD status exceptions	
**If the candidate meets this criteria:**	**Then the candidate is eligible for:**
Is admitted to the transplant hospital that registered the candidate on the waiting list and is experiencing complications of their VAD (limited to VAD complications indicated in *Policies 6.1.A-6.1.C*: life-threatening ventricular arrhythmia, hemolysis, pump thrombosis, right heart failure, device infection, mucosal bleeding, and aortic insufficiency). Note single-ventricle VADs are currently classified into status 2 in policy^5^	Status 1 exception
Is admitted to the transplant hospital that registered the candidate on the waiting list and meets *any* of the following: • Supported by *on*e of the following: • A continuous infusion of at least one high-dose intravenous inotrope: ▪ Dobutamine greater than or equal to 7.5 mcg/kg/min ▪ Milrinone greater than or equal to 0.50 mcg/kg/min ▪ Epinephrine greater than or equal to 0.02 mcg/kg/min • A continuous infusion of at least two intravenous inotropes: ▪ Dobutamine greater than or equal to 3 mcg/kg/min ▪ Milrinone greater than or equal to 0.25 mcg/kg/min ▪ Epinephrine greater than or equal to 0.01 mcg/kg/min ▪ Dopamine greater than or equal to 3 mcg/kg/min • Intolerance to maximally-tolerated inotropic dosages, as evidenced by hemodynamic instability (e.g. hypotension, vasodilation, hemodynamically unstable atrial or ventricular arrhythmias) • Mechanically ventilated Continuous monitoring of hemodynamic data, including cardiac output, with a pulmonary artery catheter or other device, is *not* required in these candidates.	Status 2 exception
Is admitted to the transplant hospital that registered the candidate on the waiting list and is experiencing complications related to their congenital heart disease (including but not limited to: protein-losing enteropathy, plastic bronchitis, or circuit thrombosis), without regard for change in the candidate’s cardiac support	Status 3 exception

All our OHT and CHLT patients are operated on by experienced congenital heart disease/heart transplant and liver transplant surgeons and patients are made status 7 if there are team members who are unavailable.

### Intraoperative Care

Proper planning, coordination and constant communication are key ingredient in successful execution of these complex procedures. Availability of team members during the entire procedure is a prerequisite to insure safety. While continuously available, the teams are rotated in and out of the operating room to reduce crowding, reduce noise and allow for focus. Rapid transfusion devices are mandatory part of equipment to ensure rapid transfusion during periods of massive blood loss. A blood bank that is adequately staffed is a key support service.

Recently, we have found it beneficial during CHLT to consider partial bypass support of the newly transplanted heart during the often long liver transplant portion of the surgery. This reduces need for blood transfusion during the liver transplant phase thus reducing exposure to additional donors, improves hemodynamic stability and allows the heart to recover from sometimes prolonged ischemia induced by cold storage during transport, dissection and implant. It is important to maintain mean arterial pressures greater than 70–75 mmHg during and after the hepatic artery anastomosis to avoid HAT (hepatic artery thrombosis). Additionally, a large proportion of patients have volume management issues and lactic academia in the operating room and are best served by having access to CRRT in the OR and in the early post-transplant period. The two perioperative challenges that characterize the CHLT procedure are vasoplegia and severe hemorrhagic coagulopathy. Vasoplegia and coagulopathy are both likely multifactorial and our approach to both of these problems is constantly evolving as we attempt to protocolize care and optimize outcomes.

Our current approach to vasoplegia includes the use of vasopressin and norepinephrine infusions with usage of methylene blue, hydroxocobalamin and/or angiotensin II in refractory cases. Angiotensin II is titratable as opposed to methylene blue which can be unpredictable in its action and is contraindicated in G6PD deficiency, renal insufficiency and in patients using SSRI medications.

Severe hemorrhage due to surgical bleeding, collaterals and coagulopathy are notable in most patients undergoing CHLT. The surgical bleeding is best controlled by ensuring adequate hemostasis during surgical dissection and utilizing peripheral cannulation if sternotomy is deemed to high risk given the retrosternal proximity of vascular or cardiac structures. It is best to control potential bleeding from AP and VV collaterals preoperatively with extensive coiling. The multifactorial coagulopathy is frequently due to prolonged cardiopulmonary bypass, the inflammatory response, liver dysfunction and consumption. All patients receive anti-fibrinolysis agents such as aminocaproic acid throughout the entirety of the case.^28^ The use of a rapid infusion device and transfusion of multiple blood products is standard in these cases. Serial ROTEM (Rotational Thromboelastometry) assessment of coagulation enables guidance of transfusion products.^29^ Typically, a prolonged period to obtain hemostasis is generally required after separation from bypass. The use of desmopressin or recombinant factors such as Factor VIIa and prothrombin complex concentrate (Kcentra or Profilnine) are frequently required for hemostasis.^30,31^ Recombinant factors must be used judiciously due to the risk of thrombosis particularly in the hepatic artery. When intractable vasoplegia, severe coagulopathy, and high inotropic support are present, a decision for early mechanical circulatory support (MCS) may need to be made as a rescue therapy. In some cases, these patients may require delayed sternal closure in order to maintain hemodynamic stability.

The biliary portion of the case is deferred and both the abdomen and chest are closed. Important intraoperative management strategies include serial point of care acid-base and arterial blood gas/electrolyte monitoring, vigilant assessment of cardiac graft function using continuous transesophageal echocardiography, aggressive management of coagulopathy using serial viscoelastic testing, and intraoperative continuous renal replacement therapy in the event of acute renal dysfunction. Bleeding is well controlled in the operating room prior to bringing the patient back to the intensive care unit.

### Post-Transplant Care and Long-Term Issues

Post-transplant care is best provided by the multi-disciplinary teams detailed earlier, however, the intensive care unit team is of foremost importance during the early post-operative period. The availability of around the clock intensivists with experience and comfort in the management of complex post-operative congenital heart disease patients is imperative. The intensive care management of the post CHLT patient is focused on hemodynamic stability, optimal ventilation, correction of metabolic derangements, and management of hemorrhagic complications. Early neurologic assessment of these patient is critical given the inherent thromboembolic risk associated with this complex surgical procedure. Careful attention to bleeding, volume status (goal CVP < 10), right ventricular function and renal function are essential in the early post-operative period. Although evidence on the early use of RRT in critical care settings remains inconclusive, we have a low threshold for initiating CRRT to minimize the risk of fluid overload and RV failure in early postoperative period.^32,33^

Continuous invasive monitoring including arterial line, CVC, PA-line and TEE are essential to achieve these goals. All members of the ICU team must be aware of the unique anatomic variation of each CHD patient. Early assessment of both the heart and liver graft function is essential. The early-OHT graft function is most often assessed by scheduled and as needed TTE, TEE and endomyocardial biopsy. Early OLT graft function is assessed by RUQ ultrasound for arterial, venous and portal blood flow as well as biochemical and synthetic markers of liver function on serial laboratory testing.

Given the anatomic heterogeneity of single ventricle patients, it is inevitable that venous and arterial connections may not be in standard locations and often require surgical innovation to create necessary connections and reconstruct structures altered by abnormal development or previous palliation procedures. Therefore, it is imperative that the congenital cardiac team remain involved and available, especially for the performance of post-transplant catheterization and biopsy procedures. For example, patients have developed areas of stenosis in the SVC, IVC and pulmonary arteries that my require intervention at a future date with angioplasty or stenting. Furthermore, congenital cardiologists can aid in post-transplant biopsies particularly when the patient has had complex venous reconstructions. Additionally, patients who underwent aortic arch reconstruction such as a Norwood may have residual issues requiring routine surveillance or stenting by an experienced congenital interventional cardiologist.

## Conclusions

Patients with failing Fontan physiology represent a heterogeneous population that requires individualized care at highly specialized centers. An immense amount of orchestration and organization is required to ensure seamless evaluation, listing, pre-transplantation optimization, operative success, and long-term survival.
